# Tentative Peptide‒Lipid Bilayer Models Elucidating Molecular Behaviors and Interactions Driving Passive Cellular Uptake of Collagen-Derived Small Peptides

**DOI:** 10.3390/molecules26030710

**Published:** 2021-01-29

**Authors:** Pathomwat Wongrattanakamon, Wipawadee Yooin, Busaban Sirithunyalug, Piyarat Nimmanpipug, Supat Jiranusornkul

**Affiliations:** 1 Laboratory for Molecular Design and Simulation (LMDS), Faculty of Pharmacy, Chiang Mai University, Chiang Mai 50200, Thailand; wipawadee.y@cmu.ac.th; 2 Department of Pharmaceutical Sciences, Faculty of Pharmacy, Chiang Mai University, Chiang Mai 50200, Thailand; busaban.s@cmu.ac.th; 3 Computational Simulation and Modelling Laboratory (CSML), Department of Chemistry, Faculty of Science, Chiang Mai University, Chiang Mai 50200, Thailand; piyarat.n@cmu.ac.th

**Keywords:** collagen-derived small peptides, membrane, molecular docking, molecular dynamics simulation, POPC, transcellular pathway

## Abstract

Collagen contains hydroxyproline (Hyp), which is a unique amino acid. Three collagen-derived small peptides (Gly-Pro-Hyp, Pro-Hyp, and Gly-Hyp) interacting across a lipid bilayer (POPC model membrane) for cellular uptakes of these collagen-derived small peptides were studied using accelerated molecular dynamics simulation. The ligands were investigated for their binding modes, hydrogen bonds in each coordinate frame, and mean square displacement (MSD) in the *Z* direction. The lipid bilayers were evaluated for mass and electron density profiles of the lipid molecules, surface area of the head groups, and root mean square deviation (RMSD). The simulation results show that hydrogen bonding between the small collagen peptides and plasma membrane plays a significant role in their internalization. The translocation of the small collagen peptides across the cell membranes was shown. Pro-Hyp laterally condensed the membrane, resulting in an increase in the bilayer thickness and rigidity. Perception regarding molecular behaviors of collagen-derived peptides within the cell membrane, including their interactions, provides the novel design of specific bioactive collagen peptides for their applications.

## 1. Introduction

Collagen is one of the major in vivo proteins that constitutes approximately 30% of the proteins and is found mainly in skin, cartilage, tendons, and bone, contributing their structures and functions [1,2]. Produced by hydrolysis of gelatin, collagen hydrolysates have been popularly used in cosmetic and food industries. Consumption of collagen hydrolysates has helpful effects; for example, increasing the moisture content of stratum corneum and bone density, improving joint pain, decreasing blood pressure, modulating the circulatory system [3], decreasing atherosclerotic plaques, anti-pruritus, and protection against photoaging [4]. Collagen contains hydroxyproline (Hyp), which is a unique amino acid [5]. A triple-helical collagen structure composes three alpha chains that includes a frequently repeating Gly-X-Y sequence, and the X and Y amino acid residues generally become Pro and Hyp. Glycylprolylhydroxyproline (Gly-Pro-Hyp), the tripeptide, whose sequence is specific for collagen [6]. Prolyl-hydroxyproline (Pro-Hyp) initiates from the Gly-Pro-Hyp sequence that has escaped complete proteolysis [5].

Gly-Pro-Hyp and Pro-Hyp show chemotactic activities on fibroblast cells, as well as peripheral blood cells as neutrophils and monocytes, which play a key role in inflammation and wound healing [7,8]. Gly-Pro-Hyp is speculated to be associated in platelet aggregation. Pro-Hyp also has stimulating activity on proliferation of mouse and human fibroblast cells and production of hyaluronic acid. Collagen tripeptides also exert osteotropic activity and improved skin quality in the mouse model and human subjects [6]. Glycyl-hydroxyproline (Gly-Hyp), collagen-derived dipeptide, stimulates activity of prolidase [EC.3.4.13.9] [9]. Prolidase particularly splits imidodipeptides with C-terminal Pro or Hyp. In the resynthesis process of collagen, this enzyme recycles Pro from imidodipeptides generally derived from degradation collagen products [10].

Regarding collagen-derived peptides, after oral collagen hydrolysate ingestion, single amino acids and di/tri-peptides were assimilated in human blood circulation. Furthermore, these di/tri-peptides can remain in the circulation for a relatively long time [11]. Pro-Hyp is a major active constituent. After the oral ingestion, Pro-Hyp can be absorbed from the gastrointestinal tract to human blood circulation. Gly-Pro-Hyp is a minor active constituent remaining in the blood, although this tripeptide is the abundant sequence. Several companies use collagenase to preserve this sequence during collagen hydrolysis, which contributes to increasing the bioavailability of the collagen-derived small peptides [6].

There are four plausible mechanisms for the transepithelial transport across the intestinal membrane of collagen-derived di- or tri-peptides: (i) proton-coupled active transport via peptide transporter (PEPT) 1 and 2, peptide histidine transporter (PHT) 1, and Ci1 (PHT2 for the human epithelial membranes) [6,12,13], (ii) a transcytotic route via brush border membrane vesicles [12], (iii) intracellular passive transport via the paracellular pathway [6], and (iv) intracellular passive transport via the transcellular pathway. However, the role of the transcellular pathway in gastrointestinal absorption of the collagen-derived peptides has not yet been completely clarified, with the transcellular mechanism for collagen-derived peptide transport across the apical side of epithelial cells still being unclear.

The objective of this study was to preliminarily investigate by construction of tentative ligand–membrane models. Therefore, model compounds of the collagen-derived small di- and tri-peptides; Gly-Pro-Hyp, Pro-Hyp, and Gly-Hyp were created to determine their possible intracellular passive transport mechanism via the transcellular pathway using MD simulation for a ligand–lipid bilayer model. Terms of molecular dynamic behaviors; the binding modes, dynamic hydrogen bonds, and mean square displacement (MSD) in the *Z* direction of these three small peptides within the 1-palmitoyl-2-oleoyl-sn-glycero-3-phosphocholine (POPC) membrane including their effect to structural properties; density (mass and electron density profiles, and area per lipid), and rigidity (root mean square deviation (RMSD)) of the membrane after the MD simulations were evaluated and compared to a control.

Molecular dynamics (MD) simulation can be applied to probe dynamical behavior of structures, interactions, and locations of different types of molecules that penetrate into cell membrane models. An initial process for the transcellular transport across the brush border membrane of the collagen-derived small peptides might be firstly through membrane interactions with specific lipid components resulting in latter dynamic movements of the membrane structure, and the molecules inside the membrane expressing by their locations. MD simulation is an outstanding tool to examine molecular interactions between membrane components and extrinsic or intrinsic molecules [14]. In this study, GPU-accelerated MD simulation in Amber14 was used to clarify the dynamical behavior including the interactions with the membrane POPC, a zwitterionic lipid with a bulky head, locations of three di-/tri-peptides; Gly-Pro-Hyp, Pro-Hyp, and Gly-Hyp. To clarify their mechanism of absorption, the single-lipid membrane model, POPC resembling the gastrointestinal membrane lipid composition [15] was used. Although single-lipid membrane models are more simplistic than multi-component lipid membrane models, the single membrane model such as the POPC model is often used to determine the effects of extrinsic molecules on a lipid bilayer by in silico [14], and also in vitro [15]. The model compounds of the collagen-derived small peptides were created to study their possible intracellular passive transport mechanism via the transcellular pathway using MD simulation for the defined model.

## 2. Results

### 2.1. Molecular Docking Model

**Figure 1:**
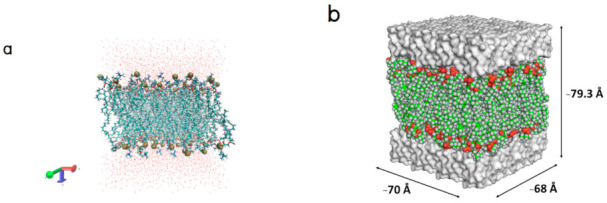
(**a**) The POPC membrane model surrounded above and below by water molecules, POPC and water molecules, and phosphorous atoms are shown in licorice, line, and van der Waals representations, respectively and (**b**) configuration of the POPC membrane. Each color represents a different component of the POPC molecule. The upper and lower white surfaces represent explicit water molecules.

Molecular docking was performed between the POPC lipid bilayer (Figure 1) and Gly-Pro-Hyp, Pro-Hyp, Gly-Hyp, and paracetamol to construct their initial binding models including solute‒membrane interactions.

For binding mode analysis as well as further dynamical structural and energy calculations, the best scored pose of each ligand‒POPC model was used. Noted is that, from the molecular docking simulation, only one small peptide molecule was inserted that could be different from the actual circumstance, the same or adjacent area of the upper leaflet could have an ability to simultaneously interact with more than one small peptide molecule. In this study, one small peptide was inserted, which represented an initial model for the next MD simulation.

### 2.2. Initial Binding Interaction

**Figure 2:**
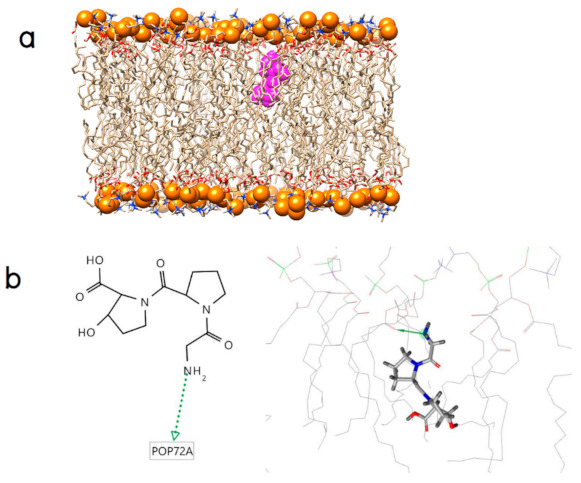
(**a**) An initial position of Gly-Pro-Hyp (magenta surface) and (**b**) Its binding modes (the green arrow represents a hydrogen bond donor).

The docked ligands were subjected to a binding mode analysis using the software LigandScout in order to understand molecular recognition at the membrane surface. From each generated computational model, the main interactions between the small peptide and the membrane was clearly observed. The binding modes of Gly-Pro-Hyp appears supported by hydrogen bond interaction between its N-terminal hydrogen atom of the glycine residue and the carbonyl oxygen in the hydrophobic tail (Figure 2a,b).

**Figure 3:**
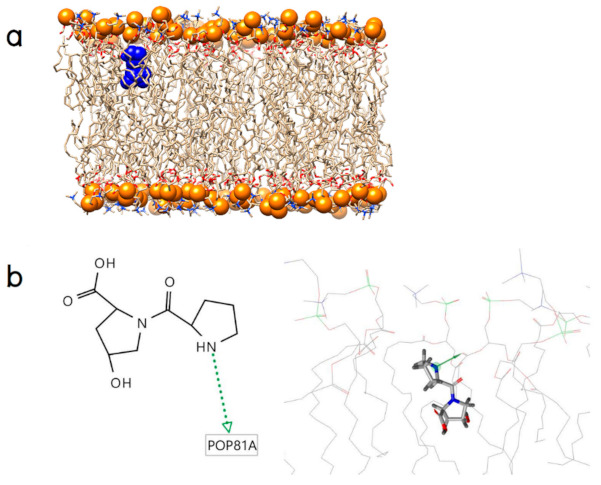
(**a**) An initial position of Pro-Hyp (blue surface) and (**b**) its binding modes (the green arrow represents a hydrogen bond donor).

Regarding Pro-Hyp, it binds on the membrane surface through one hydrogen bond which connects its N-terminal hydrogen atom in the pyrrolidine ring of the proline residue to the carbonyl oxygen in the hydrophobic tail as shown in Figure 3a,b.

**Figure 4:**
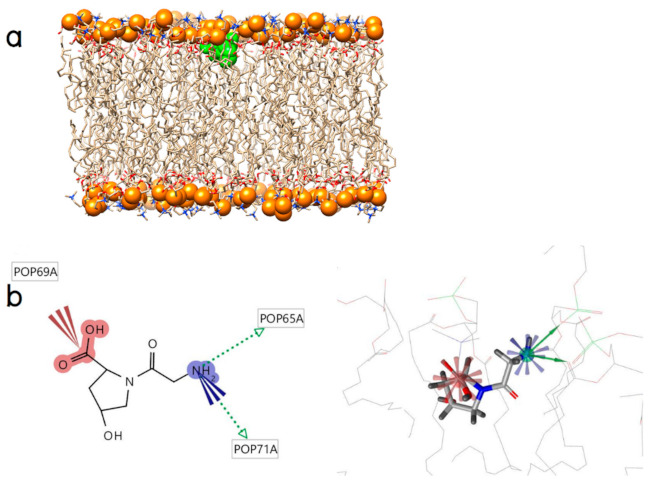
(**a**) An initial position of Gly-Hyp (green surface) and (**b**) its binding modes (the green arrows represent hydrogen bond donors, the blue spherical star represents a positive ionizable area, and the red spherical star represents a negative ionizable area).

The binding modes of Gly-Hyp appears supported by one hydrogen bond with the phosphate oxygen in the hydrophilic head generated from its N-terminal hydrogen atom of the glycine residue as a donor and one other hydrogen bond with the carbonyl oxygen in the hydrophobic tail also generated from this donor. Moreover, the positive and negative ionizable areas of Gly-Hyp at its N- and C- terminal domains, respectively, also form a charge‒charge interaction with the phosphate and choline moieties of the POPC molecules, respectively, as shown in Figure 4a,b.

**Figure 5:**
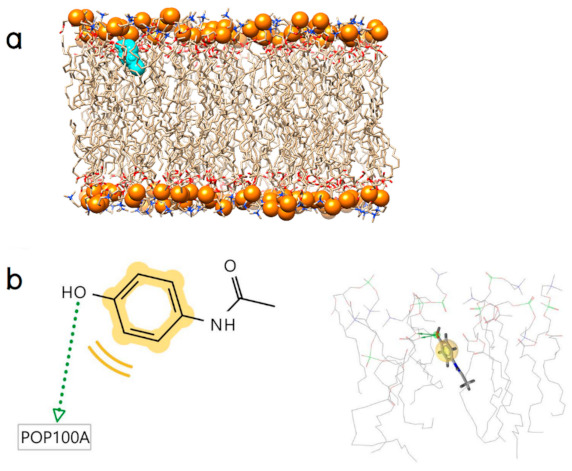
(**a**) An initial position of paracetamol (cyan surface) and (**b**) Its binding modes (the yellow sphere represents a hydrophobic feature, and the green arrows represent hydrogen bond donors).

The obtained model for the positive control paracetamol consists of two features (Figure 5a,b); (i) one hydrophobic interaction (yellow sphere) and (ii) two hydrogen bond donors (green arrows), which project to the oxygen atoms of the glycerol moiety at the hydrophilic head of the POPC molecule.

### 2.3. Dynamic Insertion Interaction and Mobility of the Small Collagen Peptides

**Figure 6:**
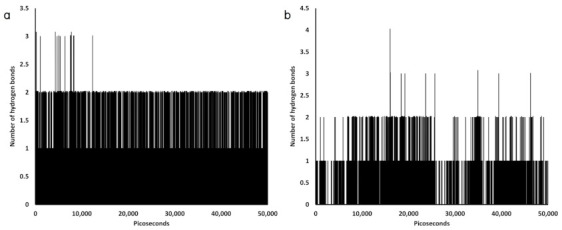

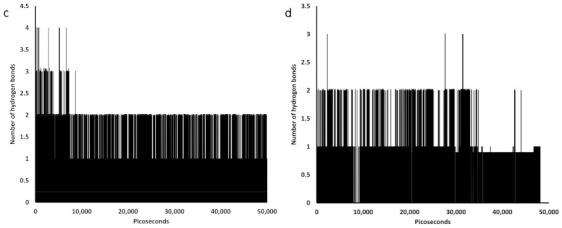
Evaluation of hydrogen bond interaction between the small collagen peptides and the POPC membrane shows the number of formed hydrogen bonds during the MD simulation; (**a**) Gly-Pro-Hyp‒POPC membrane model, (**b**) Pro-Hyp‒POPC membrane model, (**c**) Gly- Hyp‒POPC membrane model, (**d**) paracetamol‒POPC membrane model.

Evaluation of dynamic hydrogen bond interaction that contributed penetration of the small collagen peptides into the lipid bilayer (Figure 6) showed that Gly-Hyp, Gly-Pro-Hyp, and the control paracetamol formed more hydrogen bonds with the POPC membrane than Pro-Hyp, with the average numbers; 0.23256, 0.20086, 0.1745, and 0.10798 bond/picosecond, respectively.

**Figure 7:**
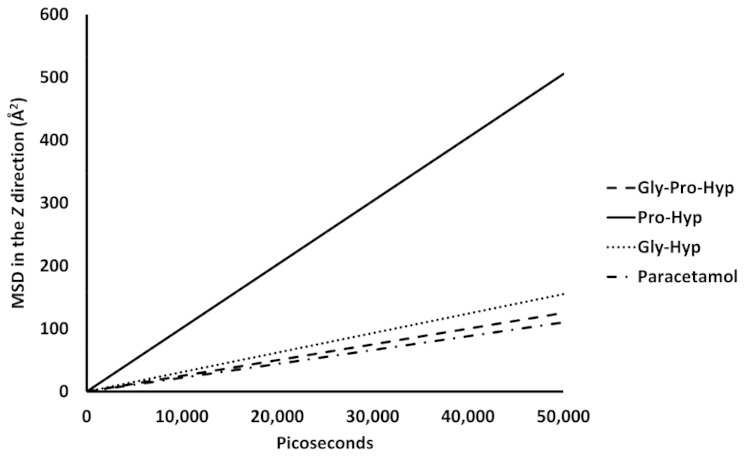
The increases in mobility of the small collagen peptides in the POPC membranes are shown by linear fits of mean square displacement (MSD) on the time scale of a picosecond. The slopes of the lines are taken to be an approximate diffusion rate.

Passive diffusion of small collagen peptides across a POPC membrane via the transcellular pathway was analyzed by mean-square displacement (MSD) as a function of time. Presence of the small collagen peptides on the POPC membrane surface was expected to interact with the POPC molecule, and initiate their mobility, as well as affect the lipid bilayer structure. The MSD of the small collagen peptides in the POPC membrane are shown in linear fits in Figure 7.

Larger slope of the linear fit reflects higher mobility in the *z* direction. Apparently, the mobility of Pro-Hyp is the highest (10.1 Å^2^/nanosecond). For all of the small peptides, the mobility is higher than that of the paracetamol‒POPC membrane model.

### 2.4. Effect of the Small Collagen Peptide on the Membrane Structure

**Figure 8:**
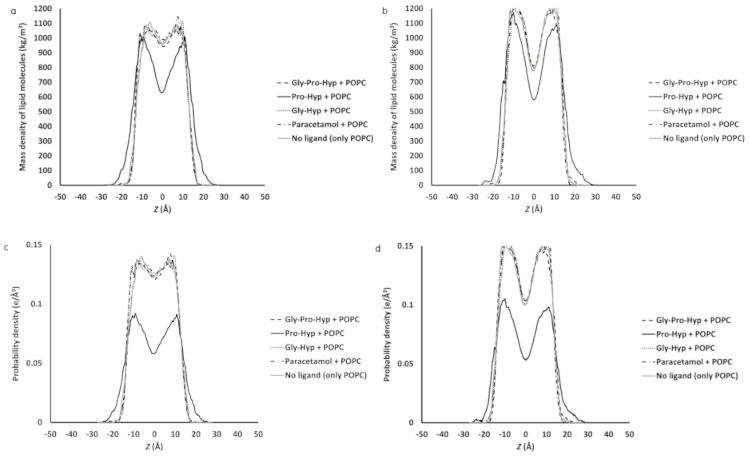
Mass density profiles of (**a**) palmitoyl groups and (**b**) oleoyl groups, and electron density profiles of (**c**) palmitoyl groups and (**d**) oleoyl groups, across the ligand‒POPC bilayer models. z = 0 corresponds to the center of the bilayer.

To determine how membrane insertion of the small collagen peptides influences distribution of the POPC molecules along the membrane *z*-axis, the mass density profile across the POPC membrane for each model that represents membrane lipid packing was calculated. The center of the bilayer (z = 0) was defined as the center of mass for the upper and lower leaflets. In comparing the mass density profiles for the different small collagen peptides, Figure 8a,b shows that as the result of Pro-Hyp, a different graph pattern compared to Gly-Pro-Hyp, Gly-Hyp, paracetamol, and pure POPC model (without any ligands) appears indicating that, near the center of the bilayer, the lipid mass density has decreased, and this is the most pronounced for the simulation for the highest mobility ligand; Pro-Hyp (Figure 6).

In Figure 8a,b, the location of the decrease suggests that Pro-Hyp deeply penetrated to the lowest density area at the center of mass and then physically pushed the hydrophobic part upward and downward for the upper and lower leaflets, respectively, causing the larger distance between the hydrophobic tails of both leaflets, and consequently resulting in the increase in thickness of the membrane (increase in the profile peak base length) while the other ligands which shallowly penetrated inside the membrane had no effects to the bulky phosphatidylcholine heads. The mass density result is consistent with the electron density distribution. Generally, both mass density and electron density profiles are the same shape [16]. From the electron density profiles, when the lower mobility molecules; Gly-Pro-Hyp, Gly-Hyp, paracetamol were simulated, an inappreciable shift of the peaks involving the phosphate moieties was observed, while the electron density distribution at the membrane center did not change or had a very slight change as shown in Figure 8c,d. It must be noted that when a molecule penetrates into a lipid membrane, the overall profile shape may be changed [16]. From the result of this study (Figure 8c,d), the electron density profile of the highest mobility peptide; Pro-Hyp was changed as compared to the pure POPC membrane that may be the result of penetration pattern. The electron density increases at the membrane interfaces and decreased at the bilayer center caused by the larger distance between the two hydrophobic parts of both layers.

**Figure 9:**
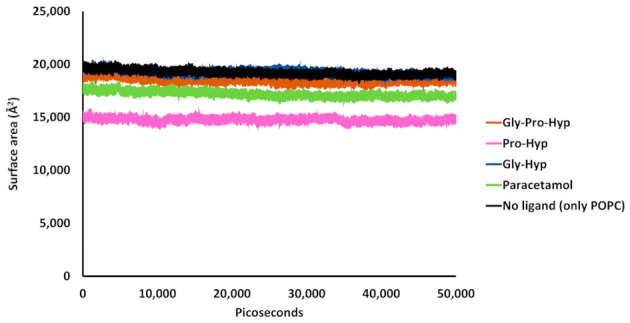
Time-series calculations of surface area (Å^2^) of the phosphatidylcholine headgroup atoms in the upper and lower leaflets of Gly-Pro-Hyp‒POPC bilayer model (orange line), Pro-Hyp‒POPC bilayer model (pink line), Gly-Hyp‒POPC bilayer model (blue line), paracetamol‒POPC bilayer model (green line), and pure POPC bilayer model (black line) during the 50,000 ps simulations.

From Figure 9, comparing the surface area of phosphatidylcholine head groups from both upper and lower leaflets in the pure POPC membrane with that of the head group in the small peptide/paracetamol -containing membranes, there is no appreciable difference among the Gly-Pro-Hyp‒POPC, Gly-Hyp‒POPC, paracetamol‒POPC models, and pure membrane model. On the contrary, the surface area of the phosphatidylcholine head groups of Pro-Hyp‒POPC bilayer model is appreciably lower than that of the pure membrane model. In the Pro-Hyp containing model, the head group atoms appear to be laterally packed, influenced by the presence of Pro-Hyp since this peptide presents a pronounced movement compared to the other molecules. In this case, the displacement of Pro-Hyp is substantially highest among the ligands (Figure 7).

**Figure 10:**
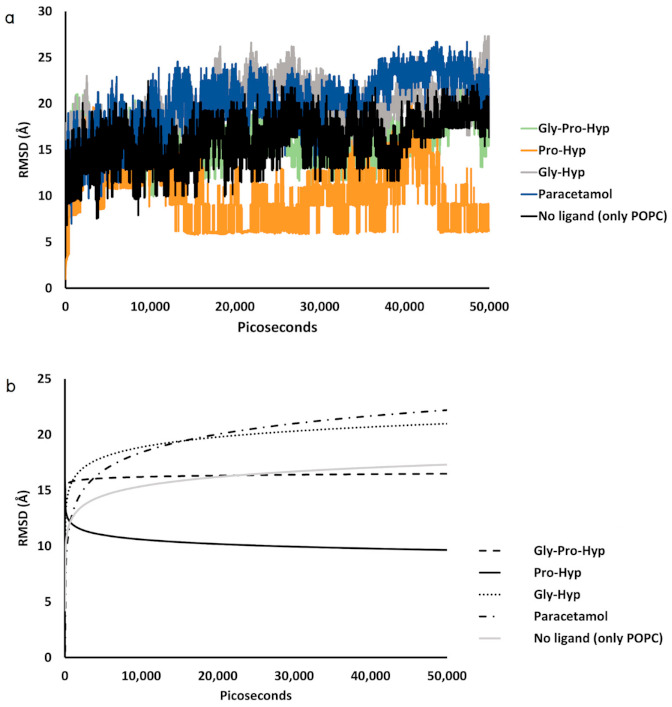
Root mean square deviation (RMSD) values of the membrane coordinates of (**a**) Gly-Pro-Hyp, Pro-Hyp, Gly-Hyp, paracetamol ‒POPC bilayer models, and pure POPC bilayer model, and (**b**) non-linear fits during the 50,000 ps simulations.

The results of time series of root mean square deviation (RMSD) with respect to an initial structure of the POPC membrane from all five peptide–POPC models and control models have been calculated to estimate the mobility. The calculated values are depicted in Figure 10, and common differences in the RMSD between the small peptides; Gly-Pro-Hyp and Gly-Hyp (including paracetamol), and pure ‒POPC membrane models are observed.

In general, an increase in membrane chain mobility is caused by interaction and penetration of a permeant in a biological membrane as well as rotation of the molecule, resulted in the higher RMSD observed. In the Gly-Hyp and paracetamol ‒POPC bilayer models, the higher RMSD than that of the pure POPC bilayer model indicates that the diffusion process of Gly-Hyp and paracetamol generally increases chain movement of the adjacent POPC molecules. In the case of Gly-Pro-Hyp, the tripeptide has a larger molecular size than other dipeptides; therefore, less free rotation of this permeant is observed with the lower effect to the lipid sidechains. Its RMSD is lower than that of the other peptides. A molecular weight difference is likely to be one that affects the diffusion of the collagen-derived small peptides across the POPC membrane. On the contrary, the RMSD of the Pro-Hyp‒POPC membrane model is appreciably lower than that of the pure POPC membrane model, indicating an increase in rigidity of the lipid bilayer when it is interacted with Pro-Hyp. Notably, this peptide produces minimal conformational change of the POPC molecules with the highest diffusion in the membrane (Figure 7).

## 3. Discussion

The Hyp-containing peptides; Pro-Hyp and Gly-Hyp (dipeptides) and Gly-Pro-Hyp (tripeptide) were demonstrated to penetrate the lipid membrane model effectively after the MD simulations compared to the positive control paracetamol.

Permeability is one of the most important fundamental properties that affects rate and extent of molecular absorption determining bioavailability of the molecule [17]. Permeability of collagen-derived peptides across a biological membrane may depend on the properties of the barrier and permeant the same as other drugs. Various parameters such as, size, charge, hydrogen bonding capacity, and lipophilicity influence permeability of a molecule across a membrane [18], and membrane properties that include type of polar head group, presence of pores, membrane thickness [16]. In peptide adsorption, the driving interactions are generally hydrophobic and electrostatic forces, and hydrogen bond interactions [19]. Determining permeability of new compounds is very important. Therefore, in an early stage of drug development, a less time-consuming and reliable method to predict compound permeability is highly required to eliminate inappropriate compounds.

Polar head groups in membrane lipids have an ability to form hydrogen bonds with other molecules. Some published studies have used simulation to investigate interaction between molecules and lipid molecules such as hydrogen bonds compared to a free membrane system. When the interacting molecule is presented, structural change of the membrane will occur [16]. From the study using Fourier transform infrared spectroscopy and extensive atomistic MD simulations by Porasso et al. [20], the interaction of unblocked amino acids including glycine which were in zwitterionic forms with a dipalmitoylphosphatidylcholine (DPPC) bilayer was investigated. Both in silico and in vitro results showed that spontaneous adsorption on the membrane surface of glycine was observed, and it afterward formed distinguishable hydrogen bonds with the hydrophilic phosphate moiety. The study using long-time MD simulations by Chen et al. [21] provides evidence that scaffolding proteins, annexins, could alter lipid membrane stability and bend the bilayer. A bent shape and concave region at the interaction interface between the annexin V trimer and the POPC/POPS (1-Palmitoyl-2-oleoyl-sn-glycero-3-phosphatidylserine) lipid bilayer molecules were observed. Three main interactions responsible for the binding were shown as calcium-bridge, hydrogen bond and hydrophobic forces. The study by Jacobs and White [22] for interfacial binding of small peptides at the bilayer interface also shows that the hydrogen bond forming by amino acid side chains is essential for insertion of the peptides.

Hydrophobic moieties of peptides do not completely contribute their insertion into membrane cores. In the case of tryptophan-containing peptides, the indole side chain of tryptophan residue contributes anchoring to an interfacial region of membranes. The indole nitrogen potentially forms hydrogen bond interactions and contributes orientation of the indole ring in the bilayer [23]. The MD simulation study by Babakhani et al. [23] shows that the tryptophan-containing peptide WL5, which contains one tryptophan residue followed by five leucine residues, spontaneously inserts into the 1,2-dimyristoyl-sn-glycero-3-phosphocholine (DMPC) membrane and then stabilizes. At the membrane interface, peptide–lipid hydrogen bonds attribute the localization. The tryptophan residue significantly contributes to the hydrogen bonds formed by its indole nitrogen.

An increase in hydrogen bonds between an interacting molecule and membrane lipid molecules points out that the molecule is spontaneously penetrated more into the membrane. In penetration through membranes, it should consider that an absorbed molecule need to cross the hydrophilic head groups of which have charges and are highly viscous, if a network of hydrogen bonds is extensively formed, permeability of the absorbed molecule may decrease [16].

The results of this study agree with the aforementioned previous studies. The glycine residues of Gly-Pro-Hyp and Gly-Hyp play an important role in hydrogen bond forming with the phosphate oxygen in the hydrophilic head and the beside carbonyl oxygen in the upper part of the hydrophobic tail of the lipid molecules in the POPC bilayer. Hydrogen bonding between an interacting molecule and biological membranes is a key factor for absorption across the membrane. The in vitro study by Srivatava et al. [24] shows that the hydrogen bond interaction affects the fluidity and permeability of the lipid bilayer model. The interacting molecule; α-tocopherol strongly binds to the dipalmitoylphosphatidylcholine (DPPC) molecules. The interaction could be a hydrogen bond between the lipid hydroxyl group and one of the two negatively charged phosphate oxygens or the glycerol oxygens. During the penetration of α -tocopherol, the molecule is brought closer to the DPPC molecule by the hydrogen bond causing changing the inter-chain distance and structure that affects the membrane fluidity, which reflects the relative motion of the membrane constituents, and then causes formation of pores near the penetration site and consequently increases the permeability of the DPPC bilayer to ascorbate ion. In similar fashion, the membrane structural change caused by the permeants, collagen-derived small peptides, could affect the membrane fluidity and consequently increase the lipid bilayer permeability to other molecules and also themselves.

Hydrogen bonding by Pro-Hyp to the POPC membrane was also observed. The N-terminal hydrogen atom in the pyrrolidine ring, a secondary amine group considered as a key pharmacophore, formed the hydrogen bond with the carbonyl oxygen of the lipid. In the case of non-N-terminal proline, which lacks this pharmacophore, traditionally, the pyrrolidine ring nitrogen of proline was never considered to be involved in hydrogen bond formation. In a polypeptide chain, the proline nitrogen atom is more basic than that of other amides. The nitrogen atom cannot participate as a hydrogen bond donor, but it is not constrained and is free to act as a hydrogen bond acceptor [25]. The recent study by Deepak and Sankararamakrishnan [25] has found the significant number of hydrogen bonds formed by proline residues that has changed this picture. The atypical and unconventional hydrogen bonds formed by the pyrrolidine ring nitrogen atoms as hydrogen bond acceptors are shown in the proteins that are necessary to support their structures.

The imino group of proline also act as a hydrogen bond donor, but it is not as effective as the amino group of other amino acids which is a primary amine [26], possibly due to having stiffness of the imino structure (planarity of the structure). In the pyrrolidine ring, it is rigidly fixed, consequently, limits conformational mobility of the N–H bond [27]. This agrees with the result of Pro-Hyp from this study that shows lesser effective in dynamic hydrogen bond interaction compared to the other peptides and paracetamol. In contrast to the other ligands, the Pro-Hyp molecule is more rigidly tilted in the bilayer. The consequently lesser hydrogen bonding leads to the particularly lower RMSD of the lipid molecules from the Pro-Hyp‒POPC bilayer model compared to the pure POPC bilayer model. The interaction between Pro-Hyp and POPC molecules increases rigidity of the bilayer. Presence of hydrogen bonding groups in a permeant as anchors contributes stabilization to its structure to bilayer sidechains, whereas the permeant, which lacks the anchors, can rotate more freely in a lipid membrane [14]. The RMSD quantity rises sharply during the initial dynamic stage of Pro-Hyp that reflects changing conformation and intermolecular spacing of the lipid molecules due to binding interaction in the adsorption stage and the initial absorption stage. After this rapid initial increase, the RMSD gradually decreases due to stiffness of the bilayer. Pro-Hyp could flip around a midplane of the lipid bilayer and further lead to localized distortions in conformation of the lipid tail, resulting in a rigid structure of the bilayer and lateral packing. Pro-Hyp shows the ability to laterally condense the POPC molecules possibly due to reducing the inter-leaflet interactions. With lateral condensation of the membrane, an increase in the bilayer thickness is observed (Figure 8). Changes in bilayer thickness can be caused by modification of interactions between upper and lower leaflets that could affect functions of biological membranes [14]. By means of this modification, Pro-Hyp could decrease interdigitation of the upper and lower membrane leaflets, which agrees with increasing the POPC bilayer thickness.

Furthermore, some studies suggest that the interaction between permeants and neighboring phospholipids laterally condenses membrane structures. It is supposed that the cholesterol’s planar ring structure (particularly the smooth alpha-face) could form attractive van der Waals interactions with the saturated fatty acyl groups of the membrane resulting in stiffening of the lipid chains, increasing the packing density, decreasing the membrane area and increasing the thickness, and consequently decreasing the membrane permeability [28,29]. An increase in membrane rigidity could result in decreasing or increasing permeability of a lipid membrane. The previous study shows that diplopterol, which is a hopanoid (a pentacyclic triterpenoid with a tertiary alcohol group), increases lipid compaction, and consequently decreases permeability on the phospholipid membranes but retains membrane fluidity and compressibility [30]. On the other hand, enhancement of lipid membrane rigidity by some substances could lead to impairment of lipid membrane integrity [31]. Furthermore, loss of integrity could result in an increase in membrane permeability, or, in other words, disruption of a membrane permeability barrier [32]. The in vitro study by Ho et al. [31] shows that polyQ35 peptide (KK-Q_35_-KK), a polyglutamine peptide increases the bilayer rigidity during adsorption and insertion resulting in only modulation of lipid membrane integrity, but not fragmentation of the membrane, by means of partial and full disruption of the intact bilayer caused by the enhanced rigidity. Moreover, polyQ35 aggregates also enhances vesicular permeability and leakage of calcein measured by time-dependent fluorescence spectroscopy. Another in vitro membrane disruption study by Devi et al. [33] shows that eugenol, a type of phenol, increases permeability of the membrane by means of the membrane disruptive action of this compound. Further studies are necessary to realize the in vivo mechanisms of absorption and other physiological effects of the collagen-derived small peptides.

## 4. Materials and Methods

### 4.1. Lipid Bilayer Structure Preparation

Initially, the planar aqueous POPC bilayer containing 128 lipid molecules (64 for each leaflet) and 4736 TIP3P water molecules were generated by CHARMM Membrane Builder GUI [34]. The models were built using the technique modified from the standard methodology from the Amber lipid force field tutorial [35]. All lipid bilayers used the TIP3P water model. Then, the charmmlipid2amber.x script was used to convert the models to Lipid14 PDB format.

### 4.2. Ligand Structure Preparation

The 3D molecular structures of the three collagen-derived di- and tri-peptides (Gly-Pro-Hyp, Pro-Hyp, and Gly-Hyp), including the positive control; paracetamol having a high intrinsic passive absorption potential [36] downloaded from PubChem [37] were prepared as docking ligands. The small peptides were minimized their energy using ChemBio3D ultra 11.0 (PerkinElmer, Waltham, MA, USA). AutoDockTools (The Scripps Research Institute, La Jolla, CA, USA) was employed to add the Gasteiger partial atomic charges to the ligands. Next, they were used as inputs for molecular docking.

### 4.3. Molecular Docking

A molecular docking approach is very useful for generating an organic molecule‒lipid membrane binding model [38]. In this study, AutoDock with the Lamarckian genetic algorithm (LGA) was used to generate binding models for the ligands to the POPC membrane. Paracetamol was docked to the POPC membrane as the positive control binding model [39,40]. The grid was created to cover the membrane upper leaflet of each model with a 126 Å cube spaced 0.375 Å. A parameter for the GA runs was set to 200. 1.0 Å root mean square deviation (RMSD) tolerance was used for docking pose clustering. The ligand pose showing the lowest final docked energy (kcal/mol) was selected as candidate binding models. Binding mode analysis of the obtained models were carried out using LigandScout [41].

### 4.4. MD Simulations

All GPU-accelerated MD simulations were carried out on the POPC bilayer that contain the docked Gly-Pro-Hyp, Pro-Hyp, and Gly-Hyp. A 50,000-picosecond (ps) MD simulation for each candidate binding model was run using Amber14 (University of California, San Francisco, CA, USA) to determine the dynamic movements of the membrane structure, and the di- and tri-peptides, as well as the positive control paracetamol inside the membranes expressing by their locations. The topology and coordinate parameters for the peptide/paracetamol‒POPC were prepared with tLeap in Amber14. The force field ff03.r1 [42] was applied for the MD simulation. ANTECHAMBER in Amber14 was used to apply force field parameters for the ligands. The GPU-accelerated MD simulation was run using GPU version of PMEMD dynamics engine of the Amber package. The MD simulation system was set following the standard methodology from the previous study [43]. The final system size of the peptide–POPC lipid membrane model are 70.0010 Å × 67.9900 Å × 79.2690 Å (X, Y, Z). VMD 1.9.2 [44] and UCSF Chimera [45] were used to visualize the results. The cpptraj module was used to calculate dynamic hydrogen bonds, MSD in the *Z* direction, mass and electron density profiles, surface area, and RMSD over the trajectories.

## 5. Conclusions

MD simulations of ligand and membrane models are widely used to study membranotropic effects including membrane packing activity, binding interactions, and locations of various compounds in biological membranes [46,47]. For example, the study using the models clarified association of epigallocatechin gallate with the lipid membrane and residing at the stable location that contributed by hydrogen bonds forming with the membrane components. The obtained results indicated the membranotropic effects of this compound [47]. This study using the in silico model of the dominant lipid; POPC of the human intestinal mucosa provided preliminary information indicating the ability of the Hyp-containing peptides which are collagen-derived dipeptides to permeate the biological membranes. The diffusion behavior of three collagen-derived small peptides; Gly-Pro-Hyp, Pro-Hyp, and Gly-Hyp across the POPC lipid bilayer was observed by the in silico methods including molecular docking, binding mode analysis, and MD simulation. Initially, molecular docking was used to generate the initial binding models of these small peptides and the positive control, paracetamol on the lipid surface. The permeants was initially bound into the polar head groups and the upper part of the hydrophobic tail of the POPC membrane, and afterwards moved underneath the hydrophobic tails of the upper leaflet to the lower leaflet of the bilayer. MD simulation ran over a period of 50,000 ps shows that the ligands prefers to interact with the POPC molecules mainly through hydrogen bonds. Pro-Hyp penetrates rather deep to the inner layer and exerts a condensing effect on the POPC bilayer. The small peptides can permeate through the lower leaflet of the POPC bilayer with relatively higher mobility rates than that of paracetamol. The results of this study are indicators of a possible mechanism of some collagen-derived small peptides penetrating across the plasma membrane of the gastrointestinal tract as intracellular passive transport via the transcellular pathway. This evaluation is presently a key component in design and development for collagen-derived small peptides.

## Data Availability

All data presented in this study are available in the article.

## Sample Availability

Samples of the compounds are not available from the authors.

## Abbreviations

AMBER	Assisted model building with energy refinement
CHARMM	Chemistry at Harvard Macromolecular Mechanics
DMPC	1,2-Dimyristoyl-sn-glycero-3-phosphocholine
DPPC	Dipalmitoylphosphatidylcholine
GA	Genetic algorithm
Gly	Glycine
Gly-Hyp	Glycyl-hydroxyproline
Gly-Pro-Hyp	Glycylprolylhydroxyproline
GPU	Graphics processing unit
GUI	Graphical user interface
Hyp	Hydroxyproline
K	Lysine
kcal/mol	Kilocalorie per mole
LGA	Lamarckian genetic algorithm
MD	Molecular dynamics
MSD	Mean square displacement
PDB	Protein data bank
PEPT	Peptide transporter
PHT	Peptide histidine transporter
PMEMD	Particle mesh Ewald molecular dynamics
POPC	1-palmitoyl-2-oleoyl-sn-glycero-3-phosphocholine
Pro	Proline
Pro-Hyp	Prolyl-hydroxyproline
Ps	Picoseconds
Q	Glutamine
RMSD	Root mean square deviation
TIP3P	Transferable intermolecular potential with 3 points
UCSF	The University of California, San Francisco
VMD	Visual molecular dynamics
Å	Angstrom
